# Molecular Mechanisms of Tumor Progression and New Therapeutic Strategies for Urological Cancers

**DOI:** 10.3390/ijms242115795

**Published:** 2023-10-31

**Authors:** Vicenç Ruiz de Porras, Albert Font

**Affiliations:** 1 CARE Program, Germans Trias i Pujol Research Institute (IGTP), 08916 Badalona, Spain; 2 Catalan Institute of Oncology, Badalona Applied Research Group in Oncology (B·ARGO), 08916 Badalona, Spain; 3 Grup de Recerca en Toxicologia (GRET), Unitat de Toxicologia, Departament de Farmacologia, Toxicologia i Química Terapèutica, Facultat de Farmàcia i Ciències de l’Alimentació, Universitat de Barcelona, Avda. Joan XXIII s/n, 08028 Barcelona, Spain; 4 Medical Oncology Department, Catalan Institute of Oncology, 08916 Badalona, Spain

Urological cancer encompasses a diverse range of tumors, including bladder, prostate, renal, upper urinary tract, and germ cell tumors. Over the past four decades, the incidence of urological cancers has increased significantly, making them a major global health concern. Strikingly, each of these urological cancers is consistently among the top ten most prevalent malignancies, contributing significantly to the overall cancer burden. This surge in incidence is particularly pronounced in men, where urological tumors collectively account for up to 33% of all reported malignancies [1]. This epidemiological reality underscores the urgent need to increase our understanding of these cancers, each of which is characterized by distinct pathogenetic mechanisms and requires varied diagnostic and therapeutic approaches. Despite considerable advances in the treatment options for urological tumors, including the integration of novel approaches such as immunotherapy and targeted therapies [2], an improved understanding of the molecular mechanisms of tumor progression and therapy resistance will help us develop novel treatment strategies and predict drug response, thus aiding our progress toward precision medicine. In this Special Issue, we have compiled a series of original research articles and a comprehensive review that explore various aspects of our emerging knowledge of molecular biology, novel mechanisms of therapy resistance, predictive biomarkers, and cutting-edge therapeutic strategies in the field of urological cancers.

In the quest for improved treatments for urological tumors, research on renal cell carcinoma (RCC) has taken a prominent role. RCC is the most prevalent kidney cancer in adults, with clear-cell RCC (ccRCC) being the most commonly diagnosed [1]. Recent studies have demonstrated that several genetic polymorphisms play a pivotal role in the prognosis of RCC and can significantly influence patient outcomes following treatment [3]. Andrzejczak and colleagues have investigated the role of polymorphisms of mucin-domain-containing-3 (TIM-3) and its ligand, galectin-9 (LGALS9), in terms of susceptibility to ccRCC and in terms of overall patient survival (OS). Their results showed an association between some single-nucleotide polymorphisms (SNPs) and clinical features and between the TIM-3 genetic variant rs1036199 and a shorter OS. Their findings provide valuable insights into the genetic factors that may affect both the risk of ccRCC and patient outcomes [4].

Over the last 15 years, significant progress has been made in the field of targeted therapy for metastatic RCC (mRCC) [5]. This progress has been achieved primarily through the introduction of tyrosine kinase inhibitors (TKIs) and immune checkpoint inhibitors (ICIs), either as stand-alone treatments or in combination with TKIs [6]. However, patients with mRCC, particularly those resistant to standard therapies such as sunitinib, are in urgent need of innovative approaches. One such approach centers around cabozantinib, a TKI that has shown promise in impeding the growth of sunitinib-resistant cell lines by targeting MET and AXL overexpression [7]. The findings presented here by Zaccagnino and colleagues shed light on the complex interplay between TKIs and resistance mechanisms in mRCC. They observed that cabozantinib appeared to be cell-line-specific, highlighting patient heterogeneity and the potential influence of alternative pathways in drug resistance. Thus, in the 786-O/S cells, which had developed resistance to sunitinib, cabozantinib exhibited less growth inhibition than in the non-sunitinib-resistant 786-O/WT cells, underscoring the inherent challenges in the effective treatment of sunitinib-resistant mRCC. Moreover, in 786-O/S cells, cabozantinib did not significantly reduce the phosphorylation levels of MET and AXL. Interestingly, among the alternative resistance mechanisms to TKIs, the activation of the Src-FAK signaling pathway seems to counteract the effects of cabozantinib, suggesting its role as an early indicator of the therapy response [8].

In another study on resistance to systemic therapy in RCC, Kowalewski and colleagues found that toll-interacting protein (TOLLIP), a regulator of autophagy and chemoresistance, may play a role in overcoming resistance. Interestingly, the TOLLIP expression was significantly higher in normal adjacent tissue than in primary RCC or mRCC tumor tissue, indicating its potential influence on cancer progression. Furthermore, the positive correlation observed between a higher TOLLIP expression and a shorter OS suggests that targeting TOLLIP may enhance the efficacy of systemic therapies in RCC. Mechanistically, TOLLIP acts as a ubiquitin-LC3 adaptor in autophagy-related signaling. This connection provides valuable insights into how TOLLIP may augment autophagy and potentially render RCC less susceptible to therapeutic interventions [9]. In fact, this study is in line with other studies demonstrating that autophagy plays a significant role in RCC, with many autophagy-related proteins serving as prognostic markers [10].

It is clear that within the ever-evolving landscape of RCC, the search for reliable diagnostic markers is a long-standing clinical challenge. Long non-coding RNAs (lncRNAs) have emerged as promising biomarkers, since they modulate genes at various levels, from epigenetics to post-transcription [11]. In line with other recent publications [12,13], the comprehensive review by Rysz and colleagues describes the roles of specific lncRNAs in RCC and suggests their potential as diagnostic and prognostic markers. Understanding the intricate mechanisms by which lncRNAs influence the disease progression in RCC is essential for the development of targeted therapies that can improve patient outcome [14].

Bladder cancer (BC), the tenth most common cancer worldwide [1], is a heterogeneous disease with a highly variable prognosis, which is mainly dependent on the pathological stage, defined by the level of tumor infiltration of the bladder wall, the regional pelvic node involvement, and the presence of distant metastases [15]. Tumors invading the detrusor muscle, known as muscle-invasive BC (MIBC), account for 20% of newly diagnosed cases of BC and for 15–20% of cases resulting from progression of non-MIBC (NMIBC) to MIBC [16]. In recent years, significant efforts have been dedicated to developing a molecular classification of MIBC, with an ultimate goal of providing a more precise understanding of the biology of the disease and facilitating tailored treatment. Kamoun and colleagues report here on a collaborative effort to integrate previously published classifications, resulting in six consensus-based molecular subtypes for MIBC [17].

In addition, our growing understanding of the molecular profile of BC can help elucidate the differences between MIBC and NMIBC. Perez-Montiel and colleagues have examined the genetic landscape of Mexican BC patients, a hitherto understudied population, and found a high frequency of mutations in TP53 and KMT2D, along with gains in 11q15.5 and 19p13.11-q12 and losses in 7q11.23. Furthermore, STAG2 mutations and 1q11.23 deletions were associated with NMIBC and a low histologic grade, which offers fresh perspectives for classifying BC subtypes [18].

Bacillus Calmette–Guérin (BCG) is currently the most effective intravesical immunotherapy for high-risk NMIBC, as it can prevent disease recurrence and progression [15]. However, a relatively high rate of BCG failure, as well as the appearance of side effects, emphasize the need for effective alternative treatment strategies, such as ICIs [19]. Recent evidence has demonstrated that BCG treatment leads to changes in the tumor microenvironment, with an increase in genes regulating immune checkpoints and a significant reduction in neoantigens, which suggests the potential effectiveness of combining ICIs with BCG in relapsed NMIBC tumors [20]. Domingos-Pereira and colleagues have characterized the immune cell infiltration in a mouse orthotopic MB49 bladder tumor model. Their findings revealed a significant increase in CD45+ immune cells over time, with a predominant presence of immunosuppressive myeloid cells, particularly monocytic myeloid-derived suppressor cells (M-MDSC) and polymorphonuclear (PMN)-MDSC. The study also highlights the correlation between PD-L1 tumor expression and the efficacy of anti-PD-1 treatment, which validates the orthotopic MB49 bladder-tumor model for designing novel therapeutic strategies. Additionally, the analysis of the chemoattractant expression suggests several potential targets, such as CCL8, CCL12, CCL9, CCL6, CXCL2, CXCL5, CXCL12, and C5/C5a antagonists, for decreasing myeloid-suppressive cells. However, the complexity of chemokine crosstalk indicates that targeting multiple chemokines may be necessary to achieve anti-tumor efficacy. This insight opens new avenues for understanding the immune landscape of BC and offers potential targets for modulating the tumor microenvironment, paving the way for enhanced treatment outcomes [21].

On a different note, but still within the field of BC treatment, Tohi and colleagues have demonstrated that BC cell lines treated with D-allose, a rare type of sugar, showed reduced cell viability and increased intracellular oxidative stress levels. Moreover, in a xenograft mouse model, oral administration of D-allose inhibited tumor growth with no adverse effects, suggesting that D-allose could be a potential therapeutic compound for the treatment of BC, possibly acting through the upregulation of thioredoxin-interacting protein (TXNIP) and a subsequent increase in intracellular reactive oxygen species (ROS) [22]. While it is true that in recent years, the antitumor role of D-allose, particularly in combination with other therapies [23], has sparked interest within the scientific community, to date, as the authors point out, preclinical results are still preliminary. Thus, the potential of D-allose as an anticancer agent holds promise, but it must be acknowledged that comprehensive research and clinical trials are required to thoroughly assess its safety and effectiveness in a clinical setting.

Prostate cancer (PC) is the most common cancer in men and was the fifth most common cause of cancer deaths in men worldwide in 2020 [1]. The development of castration-resistant prostate cancer (CRPC) is a pressing concern for patients with high-risk, locally advanced, or metastatic prostate cancer who have undergone androgen deprivation therapy (ADT) [24]. Several studies have shown that epigenetic dysregulation is a driving mechanism in PC progression, primarily through the androgen receptor, which plays a pivotal role in CRPC development through epigenetic modification [25]. Epigenetic alterations take place in the earliest phases of PC, indicating their important function in PC initiation, and persist throughout disease progression and metastasis [26]. Orea and colleagues have demonstrated that the epigenetic silencing of claudin-3 (CLDN3) is a common occurrence in CRPC and is associated with increased cellular invasion. Moreover, the loss of CLDN3 expression is linked to a shorter disease-free survival and time before clinical progression in PC patients. This finding raises the prospect of CLDN3 as a potential molecular marker capable of distinguishing aggressive from indolent prostate tumors [27].

Finally, the cell stress response, managed by the heat shock factor (HSF)–heat shock protein (HSP) system, plays a vital function in the adaptation of cancer cells to environmental stress. SCAN domain transcription factors (SCAND-TF), including SCAND1 and SCAND2, are involved in repressing the stress response in cancer by co-repressing target genes. Sheta and colleagues have demonstrated that heat stress induces the expression of SCAND1, SCAND2, and MZF1, which are bound to the HSP90 gene promoter regions in PC cells, suggesting that the stress-inducible SCAN-TFs act as a feedback system, suppressing an excessive stress response and inhibiting cancer progression [28]. Interestingly, a recent study also demonstrated that SCAND1 overexpression leads to the reversal of the epithelial-to-mesenchymal transition (EMT), a well-known process involved in tumor progression and therapy resistance, and thereby inhibits PC cell proliferation and migration in vitro and in vivo [29].

Together, the contributions to this Special Issue, which is currently in its second edition, span a wide spectrum of topics, all of which are poised to advance our understanding of urological oncology and inform the development of more effective clinical approaches. From elucidating the intricacies of molecular pathways driving urological cancers to identifying diagnostic biomarkers and innovative strategies aimed at overcoming resistance to current therapies, the content herein promises to be an invaluable resource for researchers, clinicians, and healthcare professionals seeking to stay at the forefront of this rapidly evolving field.

## References

[1] Sung H., Ferlay J., Siegel R.L., Laversanne M., Soerjomataram I., Jemal A., Bray F. (2021). Global Cancer Statistics 2020: GLOBOCAN Estimates of Incidence and Mortality Worldwide for 36 Cancers in 185 Countries. CA Cancer J. Clin..

[2] Jackson-Spence F., Young M., Sweeney C., Powles T. (2023). Top advances of the year: Genitourinary cancer. Cancer.

[3] Shiota M., Miyake H., Takahashi M., Oya M., Tsuchiya N., Masumori N., Matsuyama H., Obara W., Shinohara N., Fujimoto K. (2023). Effect of genetic polymorphisms on outcomes following nivolumab for advanced renal cell carcinoma in the SNiP-RCC trial. Cancer Immunol. Immunother..

[4] Andrzejczak A., Tupikowski K., Tomkiewicz A., Malkiewicz B., Ptaszkowski K., Domin A., Szydelko T., Karabon L. (2023). The Variations’ in Genes Encoding TIM-3 and Its Ligand, Galectin-9, Influence on ccRCC Risk and Prognosis. Int. J. Mol. Sci..

[5] Ravi P., Bakouny Z., Schmidt A., Choueiri T.K. (2020). Novel Therapeutic Approaches and the Evolution of Drug Development in Advanced Kidney Cancer. Cancer J..

[6] Lalani A.A., Heng D.Y.C., Basappa N.S., Wood L., Iqbal N., McLeod D., Soulieres D., Kollmannsberger C. (2022). Evolving landscape of first-line combination therapy in advanced renal cancer: A systematic review. Ther. Adv. Med. Oncol..

[7] Choueiri T.K., Escudier B., Powles T., Mainwaring P.N., Rini B.I., Donskov F., Hammers H., Hutson T.E., Lee J.L., Peltola K. (2015). Cabozantinib versus Everolimus in Advanced Renal-Cell Carcinoma. N. Engl. J. Med..

[8] Zaccagnino A., Vynnytska-Myronovska B., Stockle M., Junker K. (2023). An In Vitro Analysis of TKI-Based Sequence Therapy in Renal Cell Carcinoma Cell Lines. Int. J. Mol. Sci..

[9] Kowalewski A., Jaworski D., Borowczak J., Maniewski M., Szczerbowski K., Antosik P., Durslewicz J., Smolinska M., Ligmanowska J., Grzanka D. (2022). TOLLIP Protein Expression Predicts Unfavorable Outcome in Renal Cell Carcinoma. Int. J. Mol. Sci..

[10] He Y.H., Tian G. (2020). Autophagy as a Vital Therapy Target for Renal Cell Carcinoma. Front. Pharmacol..

[11] Beylerli O., Gareev I., Sufianov A., Ilyasova T., Guang Y. (2022). Long noncoding RNAs as promising biomarkers in cancer. Noncoding RNA Res..

[12] Chao X., Wang P., Ma X., Li Z., Xia Y., Guo Y., Ge L., Tian L., Zheng H., Du Y. (2021). Comprehensive analysis of lncRNAs as biomarkers for diagnosis, prognosis, and treatment response in clear cell renal cell carcinoma. Mol. Ther. Oncolytics.

[13] Gao X., Zhang H., Zhang C., Li M., Yu X., Sun Y., Shi Y., Zhang H., He X. (2023). The emerging role of long non-coding RNAs in renal cell carcinoma progression and clinical therapy via targeting metabolic regulation. Front. Pharmacol..

[14] Rysz J., Konecki T., Franczyk B., Lawinski J., Gluba-Brzozka A. (2022). The Role of Long Noncoding RNA (lncRNAs) Biomarkers in Renal Cell Carcinoma. Int. J. Mol. Sci..

[15] Kamat A.M., Hahn N.M., Efstathiou J.A., Lerner S.P., Malmstrom P.U., Choi W., Guo C.C., Lotan Y., Kassouf W. (2016). Bladder cancer. Lancet.

[16] Ruiz de Porras V., Pardo J.C., Etxaniz O., Font A. (2022). Neoadjuvant therapy for muscle-invasive bladder cancer: Current clinical scenario, future perspectives, and unsolved questions. Crit. Rev. Oncol. Hematol..

[17] Kamoun A., de Reynies A., Allory Y., Sjodahl G., Robertson A.G., Seiler R., Hoadley K.A., Groeneveld C.S., Al-Ahmadie H., Choi W. (2020). A Consensus Molecular Classification of Muscle-invasive Bladder Cancer. Eur. Urol..

[18] Perez-Montiel M.D., Cerrato-Izaguirre D., Sanchez-Perez Y., Diaz-Chavez J., Cortes-Gonzalez C.C., Rubio J.A., Jimenez-Rios M.A., Herrera L.A., Scavuzzo A., Meneses-Garcia A. (2023). Mutational Landscape of Bladder Cancer in Mexican Patients: KMT2D Mutations and chr11q15.5 Amplifications Are Associated with Muscle Invasion. Int. J. Mol. Sci..

[19] Kodera A., Mohammed M., Lim P., Abdalla O., Elhadi M. (2023). The Management of Bacillus Calmette-Guerin (BCG) Failure in High-Risk Non-muscle Invasive Bladder Cancer: A Review Article. Cureus.

[20] Su F., Liu M., Zhang W., Tang M., Zhang J., Li H., Zou L., Zhang R., Liu Y., Li L. (2022). Bacillus Calmette-Guerin Treatment Changes the Tumor Microenvironment of Non-Muscle-Invasive Bladder Cancer. Front. Oncol..

[21] Domingos-Pereira S., Sathiyanadan K., Polak L., Haefliger J.A., Schmittnaegel M., Ries C.H., Jichlinski P., Roth B., Derre L., Nardelli-Haefliger D. (2022). Tumor-Microenvironment Characterization of the MB49 Non-Muscle-Invasive Bladder-Cancer Orthotopic Model towards New Therapeutic Strategies. Int. J. Mol. Sci..

[22] Tohi Y., Taoka R., Zhang X., Matsuoka Y., Yoshihara A., Ibuki E., Haba R., Akimitsu K., Izumori K., Kakehi Y. (2022). Antitumor Effects of Orally Administered Rare Sugar D-Allose in Bladder Cancer. Int. J. Mol. Sci..

[23] Khajeh S., Ganjavi M., Panahi G., Zare M., Zare M., Tahami S.M., Razban V. (2023). D-allose: Molecular Pathways and Therapeutic Capacity in Cancer. Curr. Mol. Pharmacol..

[24] Davies A., Conteduca V., Zoubeidi A., Beltran H. (2019). Biological Evolution of Castration-resistant Prostate Cancer. Eur. Urol. Focus.

[25] Ruggero K., Farran-Matas S., Martinez-Tebar A., Aytes A. (2018). Epigenetic Regulation in Prostate Cancer Progression. Curr. Mol. Biol. Rep..

[26] Pardo J.C., Ruiz de Porras V., Gil J., Font A., Puig-Domingo M., Jorda M. (2022). Lipid Metabolism and Epigenetics Crosstalk in Prostate Cancer. Nutrients.

[27] Orea M.J., Angulo J.C., Gonzalez-Corpas A., Echegaray D., Marva M., Lobo M.V.T., Colas B., Ropero S. (2023). Claudin-3 Loss of Expression Is a Prognostic Marker in Castration-Resistant Prostate Cancer. Int. J. Mol. Sci..

[28] Sheta M., Yoshida K., Kanemoto H., Calderwood S.K., Eguchi T. (2023). Stress-Inducible SCAND Factors Suppress the Stress Response and Are Biomarkers for Enhanced Prognosis in Cancers. Int. J. Mol. Sci..

[29] Eguchi T., Csizmadia E., Kawai H., Sheta M., Yoshida K., Prince T.L., Wegiel B., Calderwood S.K. (2022). SCAND1 Reverses Epithelial-to-Mesenchymal Transition (EMT) and Suppresses Prostate Cancer Growth and Migration. Cells.

